# Supplementary material for: Mechanistic patterns and clinical implications of oncogenic tyrosine kinase fusions in human cancers
Source: Nat Commun. 2024 Jun 14;15:5110. doi: 10.1038/s41467-024-49499-0 (PMC11178778; doi:10.1038/s41467-024-49499-0)
Supplement: Supplementary file 1 — Supplementary Information [file 41467_2024_49499_MOESM1_ESM.pdf]

Supplementary Information

Mechanistic patterns and clinical implications of oncogenic tyrosine kinase fusions in human cancers

Taek-Chin Cheong[1,#,*], Ahram Jang[1,2,#], Qi Wang[1], Giulia C. Leonardi[1,3], Biagio Ricciuti[4], Joao V. Alessi[4], Alessandro Di Federico[4], Mark M. Awad[4], Maria K. Lehtinen[1], Marian H. Harris[1], Roberto Chiarle[1,5,6,*]

[1]Department of Pathology, Boston Children's Hospital and Harvard Medical School, Boston, MA 02115, USA

[2]Division of Endocrinology, Diabetes, and Metabolism, Department of Medicine, Beth Israel Deaconess Medical Center, Boston, MA 02115, USA

[3]Department of Biomedical and Biotechnological Sciences, University of Catania, 95123 Catania, Italy

[4]Lowe Center for Thoracic Oncology, Dana-Farber Cancer Institute, Boston, MA 02115, USA

[5]Department of Molecular Biotechnology and Health Sciences, University of Torino, Torino, 10126 Italy

[6]Division of Hematopathology, IEO European Institute of Oncology IRCCS, 20141, Milan, Italy

[#]These authors contributed equally

[*]Correspondence:
taekchin.cheong@childrens.harvard.edu
roberto.chiarle@childrens.harvard.edu

Supplementary information includes:

Supplementary Figures 1-10

Supplementary References

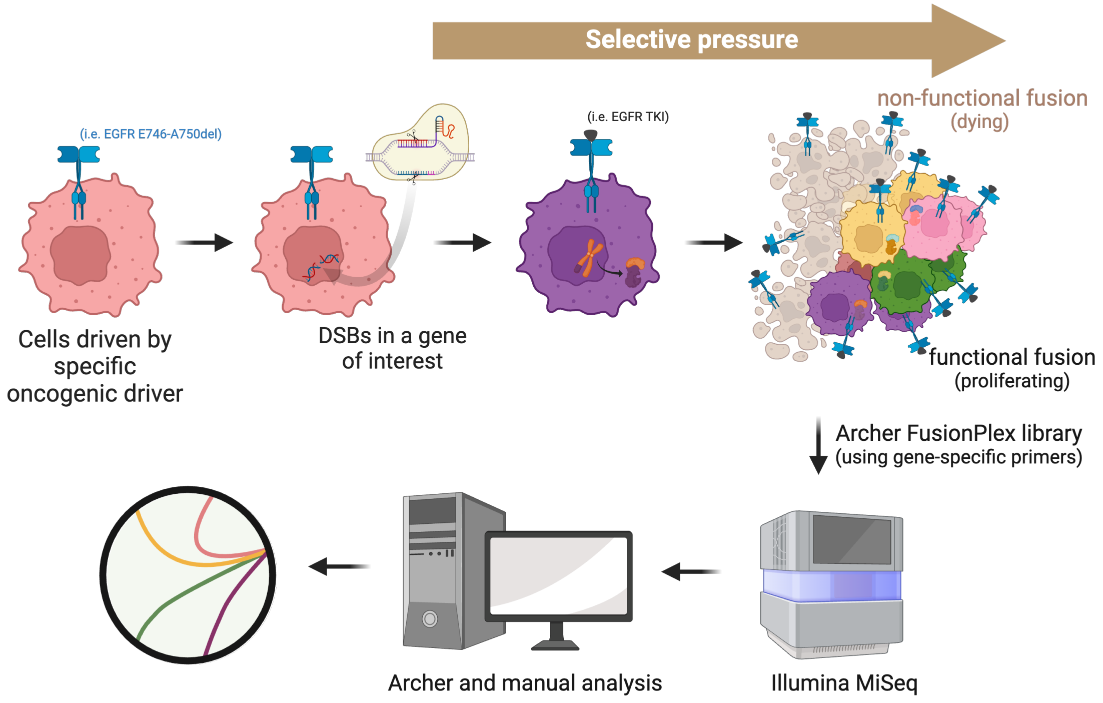

Supplementary Fig. 1. Overview of FACTS approach. Cells driven by specific oncogenic driver will be chosen, generated DNA DSBs in a gene of interest, and selected with inhibitors of the specific oncogenic driver. Library will be prepared using gene-specific primers and sequenced using illuminal MiSeq. Sequence data will be analyzed with Archer and manual analysis (see Methods). TKI, tyrosine kinase inhibitor. Schematics was created with BioRender.com.

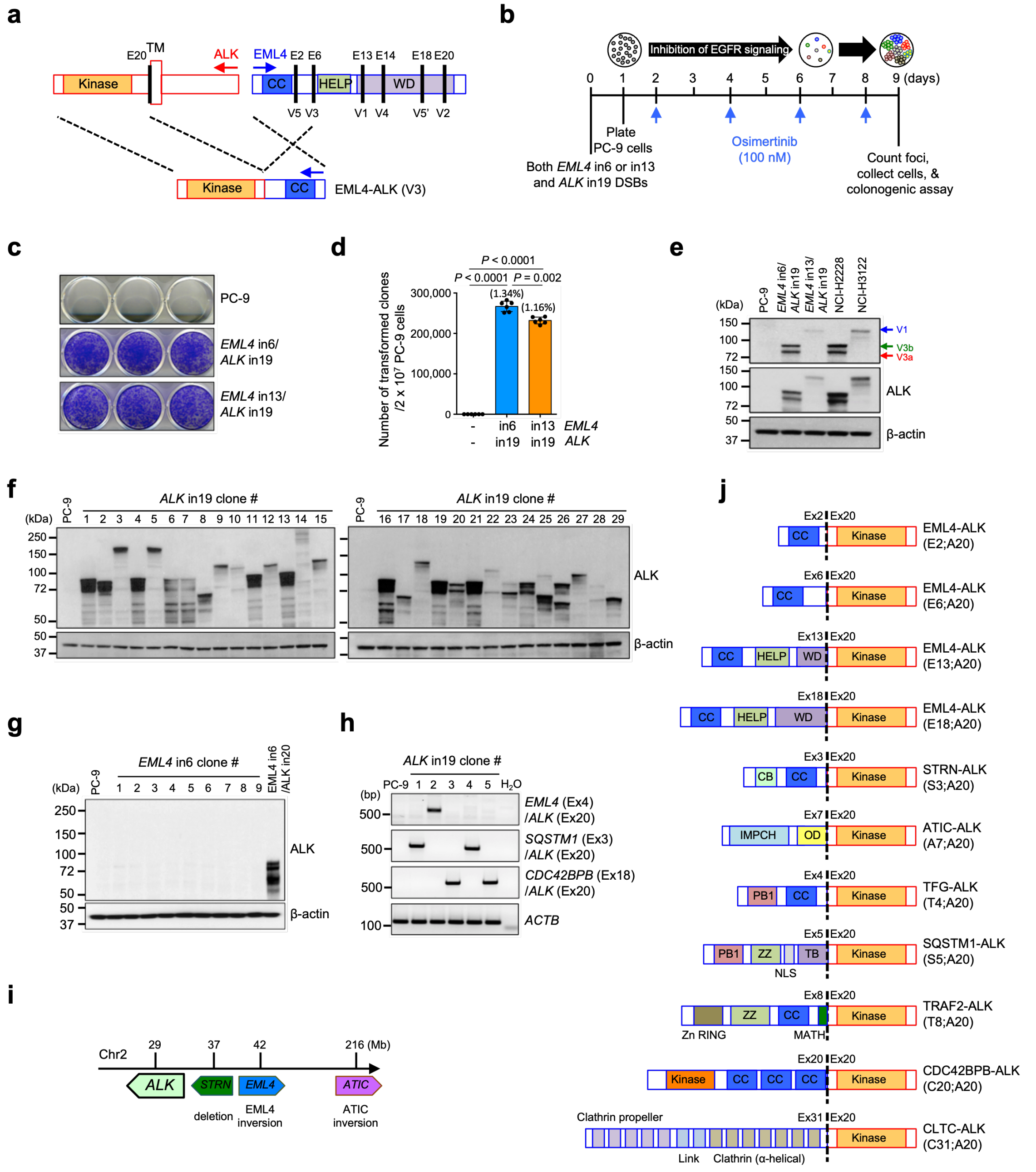

Supplementary Fig. 2. ALK fusions induce resistance to osimertinib in PC-9 cells and spontaneously formed ALK fusions drive tumor formation *in vivo*. a, Schematic representation of EML4-ALK fusion variants observed in NSCLC patients. CC, coiled-coil domain; kinase, tyrosine kinase domain; HELP, hydrophobic EML protein domain; WD, WD repeats. **b**, Timeline of the clonogenic assay. **c**, Representative images of the clonogenic assay. Similar results were obtained from n=2 independent experiments. **d**, Quantification of osimertinib-resistant clones in

cells treated as in (**b**). Data show means of n=6 biological replicates, with error bars representing ±s.e.m; significance was determined by an unpaired, two-tailed Student's *t*-test. **e**, Representative western blots of phospho-ALK and ALK in PC-9 cells expressing EML4-ALK fusion proteins. Similar results were obtained from n=2 independent experiments. NCI-H2228 and NCI-H3122 are used as positive controls for EML4-ALK E6;A20 and EML4-ALK E13;A20, respectively. **f**, Western blots for ALK fusions in osimertinib-resistant clones (n=29) where DNA DSBs were induced in intron 19 of *ALK*. **g,** Western blots of ALK in osimertinib-resistant clones (n=9) induced DNA DSBs in intron 6 of *EML4*. **h**, Detection of the indicated *ALK* fusion transcripts in osimertinib-resistant PC-9 clones. Similar results were obtained from n=2 independent experiments. **i**, Position and orientation of ALK-fusion partners located on chromosome 2. **j**, Schematic structural composition of various ALK fusion proteins. Protein domains are indicated by color and include: CC, coiled-coil domain; HELP, hydrophobic EML protein domain; WD, WD repeats; Kinase (cantaloupe), ALK tyrosine kinase domain; CB, caveolin-binding domain; IMPCH, inosine monophosphate cyclohydrolase domain; OD, oligomerization domain; PB1, Phox and Bem1 domain; ZZ, zinc finger domain; NLS, nuclear localization signal; TB, TRAF6 binding domain; Zn RING, RING-type zinc finger; MATH, meprin and TRAF homology domain; kinase (tangerine), CDC42BPB serine/threonine kinase domain. Source data are provided as a Source Data file.

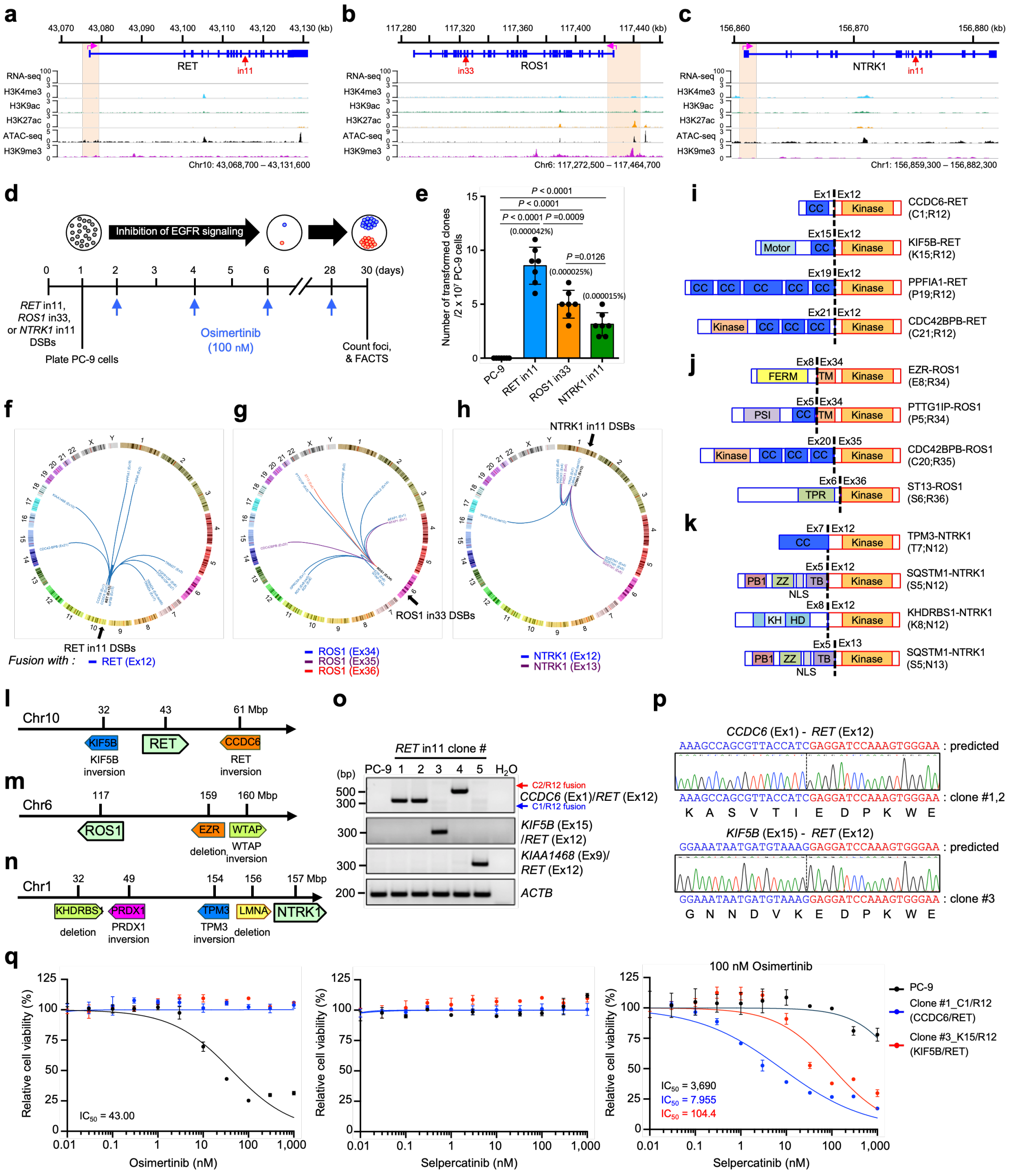

Supplementary Fig. 3. Identification and characterization of functional RET, ROS1, and NTRK1 fusions in osimertinib-resistant PC-9 cells by FACTS. a-c, RNA-seq, ChIP-seq, and ATAC-seq profiles in *RET* (**a**), *ROS1* (**b**), and *NTRK1* (**c**) genes in PC-9 cells. Transcription start site (TSS) is indicated in pink. Intron for sgRNA design is indicated in red. **d**, Experimental timeline to select osimertinib-resistant clones. **e**, Quantification of osimertinib-resistant clones in PC-9 cells in which DNA

DSBs were introduced in intron 11 of *RET*, in intron 33 of *ROS1* or intron 11 of *NTRK1* according to the scheme shown in (**d**). Data are means of n=7 biological replicates, with error bars representing ±s.e.m; significance was determined by an unpaired, two-tailed Student's *t*-test. **f-h**, Circos plots showing the genome-wide distribution of *RET* (**f**), *ROS1* (**g**), and *NTRK1* (**h**) fusion partners. Arcs represent functional rearrangements joining *RET* (exon 12), *ROS1* (exon 34, 35 or 36), or *NTRK1* (exon 12 or 13) to the indicated fusion partner. **i-k**, Schematic structural composition of example RET (**i**), ROS1 (**j**), and NTRK1 (**k**) fusion proteins identified by FACTS. Protein domains are indicated by color and include: CC, coiled-coil domain; Kinase (cantaloupe), tyrosine kinase domain; kinase (tangerine), CDC42BPB serine/threonine kinase domain; FERM, the protein 4.1R, ezrin, radixin, moesin; PSI, plexin-semaphorin-integrin; TPR, tetratricopeptide repeat; TM, transmembrane; PB1, Phox and Bem1 domain; ZZ, zinc finger domain; NLS, nuclear localization signal; TB, TRAF6 binding domain; KH, K homology domain; HD, histidine/aspartate. **l-n**, Position and orientation of *RET* (**l**), *ROS1* (**m**), and *NTRK1* (**n**) fusion partners occurring on chromosome 10 (*RET*), chromosome HMS 6 (*ROS1*), and chromosome 1 (*NTRK1*). **o**, Detection of the indicated *RET* fusion transcripts in PC-9 cells with DNA DSBs introduced in intron 11 of *RET*. Red and blue arrows indicate CCDC6-RET C2;R12 and CCDC6-RET C1;R12 fusion, respectively. Similar results were obtained from n=2 independent experiments. **p**, Chromatograms showing the fusion transcripts producing the indicated *RET* fusions. **q**, Sensitivity to osimertinib (**left**), selpercatinib (**middle**), or combination of osimertinib plus selpercatinib (**right**) in osimertinib-resistant clones. Data show means of n=3 biological replicates, with error bars representing ±s.e.m. Source data are provided as a Source Data file.

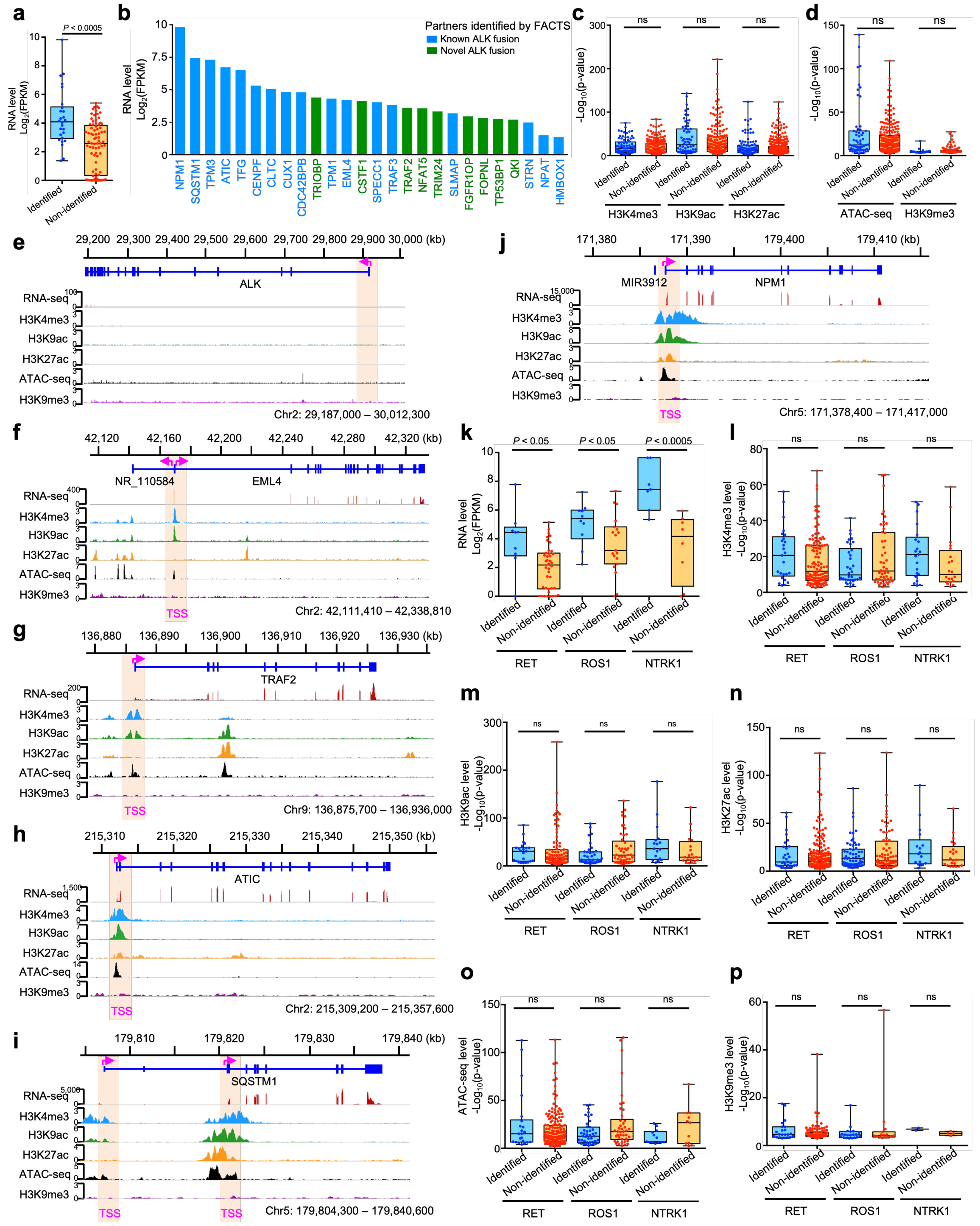

Supplementary Fig. 4. Transcription, accessibility, and chromatin features of ALK, RET, ROS1, and NTRK1 fusion partners. a, Quantification of mRNA expression by RNA-seq fragments per kilobase million (FPKM) of known *ALK* partner genes that were identified by FACTS (blue) or *ALK* partners described in patients but not

identified by FACTS in PC-9 cells (red). Each dot represents one gene. *p* value was calculated by two-tailed Welch's test. **b**, Histograms showing mRNA expression of *ALK* partner genes identified by FACTS in PC-9 cells; novel *ALK*-fusion partners never described in patients identified by FACTS are in green. **c and d,** Average H3K4me3, H3K9ac, and H3K27ac ChIP-seq values (**c**), ATAC-seq and H3H9me3 ChIP-seq values (**d**) of known ALK partner genes that were identified by FACTS (blue) vs partners that were described in patients but not identified by FACTS in PC-9 cells (red). Each dot represents one peak. *P* value was calculated by two-tailed Welch's t test. **e-j,** RNA-seq, ChIP-seq, and ATAC-seq profiles in *ALK* (**e**), *EML4* (**f**), *TRAF2* (**g**), *ATIC* (**h**), *SQSTM1* (**i**), and *NPM1* (**j**) genes. Transcription start site (TSS) is indicated in pink. **k**, Quantification of mRNA expression by RNA-seq fragments per kilobase million (FPKM) of known *RET*, *ROS1*, and *NTRK1* fusion partners were identified by FACTS (blue) vs partners that were described in patients but not identified by FACTS in PC-9 cells (red). Each dot represents one gene. *P* value was calculated by two-tailed Welch's t test. **l-n**, Average H3K4me3 (**l**), H3K9ac (**m**), or H3K27ac (**n**) ChIP-seq of known *RET*, *ROS1*, and *NTRK1* fusion genes that were identified by FACTS (blue) vs partners that were described in patients but not identified by FACTS in PC-9 cells (red). **o and p**, ATAC-seq (**o**) or H3H9me3 ChIP-seq (**p**) of known *RET*, *ROS1*, and *NTRK1* fusion genes that were identified by FACTS (blue) vs partners that were described in patients but not identified by FACTS in PC-9 cells (red). Each dot represents a peak within gene. *P* value was calculated by two-tailed Welch's t test. Source data are provided as a Source Data file.

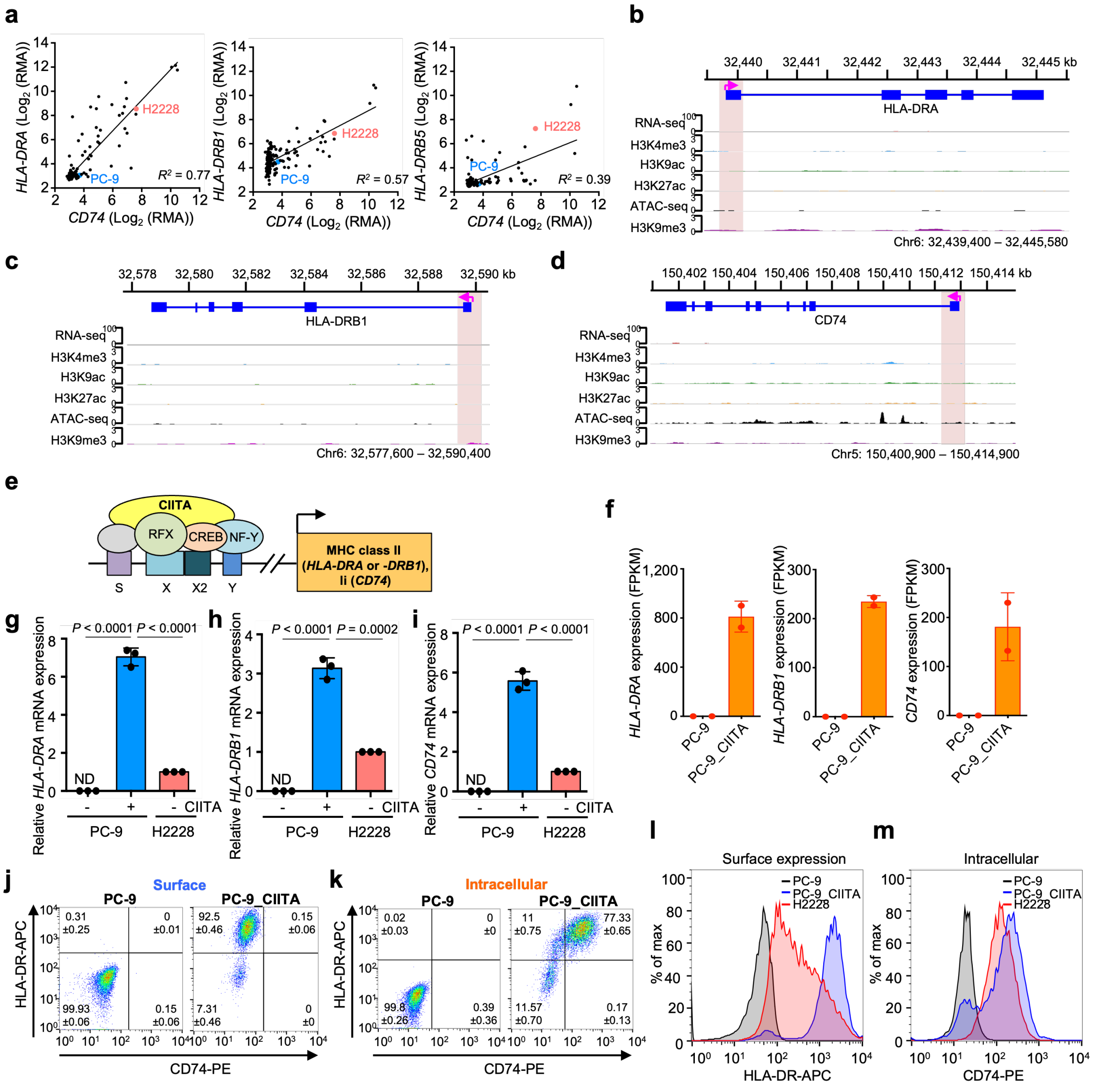

Supplementary Fig. 5. Generation of stable PC-9 cell lines expressing CIITA. a, Scatter plots showing expression value of *HLA-DRA*, *HLA-DRB1*, *HLA-DRB5*, and *CD74* in a series of lung cancer cell lines from cancer genome project (CGP). PC-9 cells and NCI-H2228 cells are indicated in blue and salmon, respectively. RMA, robust multichip average. **b-d**, RNA-seq, ChIP-seq, and ATAC-seq profiles in *HLA-DRA* (**b**), *HLA-DRB1* (**c**), and *CD74* (**d**) genes. **e**, Schematic representation of the function of CIITA on MHC class II expression. CIITA activates expression of its target genes such as MHC class II (e.g. *HLA-DRA* or *HLA-DRB1*) and Ii (*CD74*) by binding to a promoter proximal enhancer consisting of S, X, X2, and Y sequences. **f,** Analysis of messenger RNA levels of *HLA-DRA, HLA-DRB1*, and *CD74* in PC-9 expressing CIITA by RNA-seq.

Similar results were obtained from n=2 independent experiments. **g-i**, Messenger RNA levels of *HLA-DRA* (**g**), *HLA-DRB1* (**h**), and *CD74* (**i**) in PC-9 expressing CIITA were analyzed by quantitative real-time PCR (qRT-PCR). NCI-H2228 is an ALK-rearranged NSCLC lines known to express HLA-DR. Data show means of n=3 biological replicates, with error bars representing ±s.e.m; significance was determined by an unpaired, two-tailed Student's *t*-test. **j and k**, Representative pseudocolor plots showing surface (**j**) or intracellular (**k**) expression of HLA-DR and CD74 in PC-9 and CIITA-expressing PC-9 cells. Similar results were obtained from n=3 independent experiments. **l and m**, Representative histograms showing surface expression of HLA-DR (**l**) or intracellular CD74 (**m**) molecules. Data are representative of n=3 independent experiments. Source data are provided as a Source Data file.

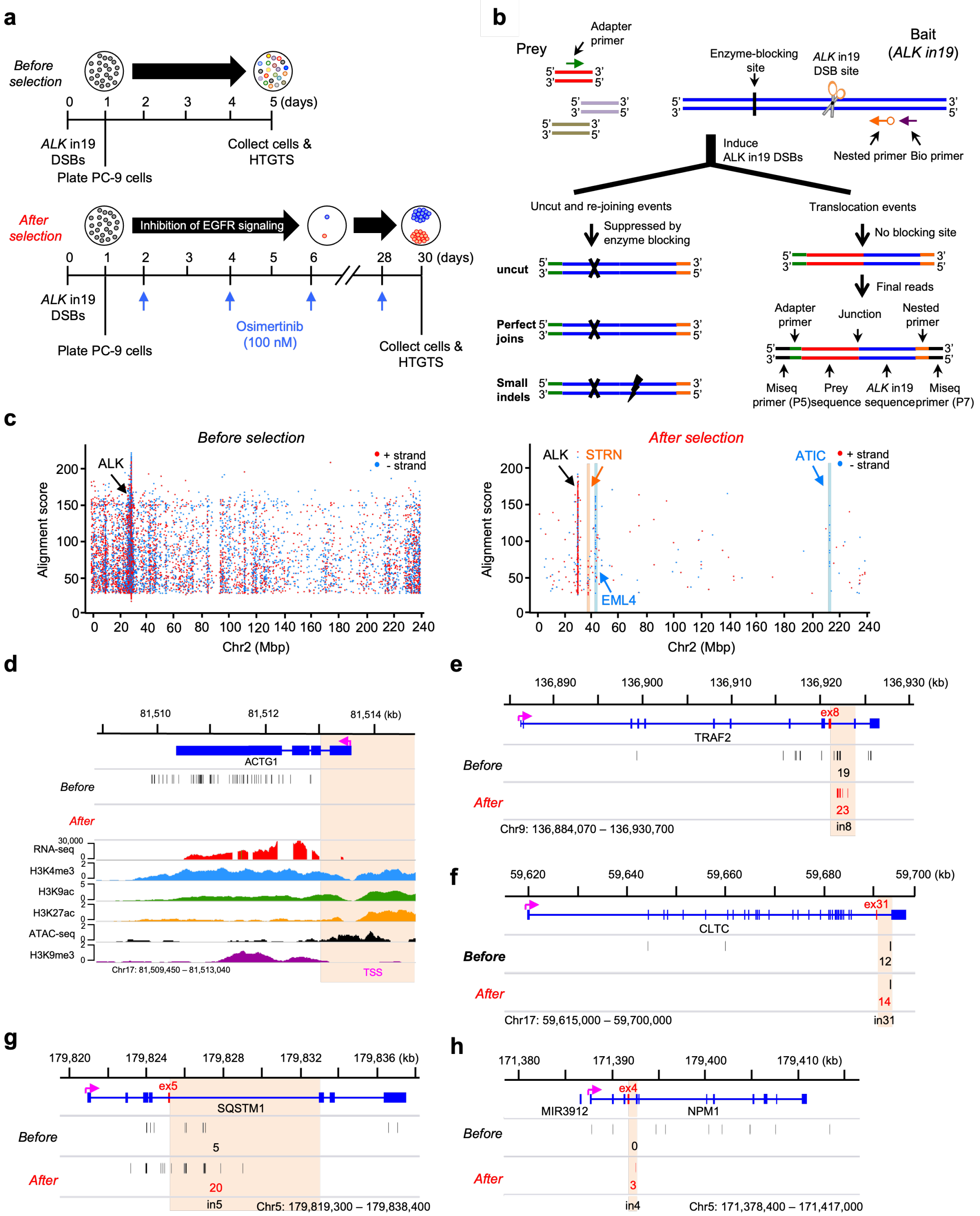

Supplementary Fig. 6. Characterization of functional ALK fusions in PC-9 cells by HTGTS. a, Experimental timeline for HTGTS analysis of *ALK* translocations. **b**, Schematic overview of HTGTS strategy to sequence *ALK* translocations by DNA DSBs generated in intron 19 of *ALK* as bait to capture genome-wide breakpoints. **c**, Rainfall plots showing DNA translocations before (**left**) and after (**right**) selection on

chromosome 2. Each dot represents a single translocation ordered on the X-axis according to its position on chromosome 2. **d**, Distribution of DNA breakpoints and histone modification marks in *ACTG1* gene. Transcription start site (TSS) is indicated in pink. **e-h**, Detailed distribution of DNA breakpoints in *TRAF2* (**e**), *CLTC* (**f**), *SQSTM1* (**g**), and *NPM1* (**h**) genes. The purple arrows indicate orientation of genes. The number of translocations in focal clusters is indicated in black and red for before and after selection, respectively.

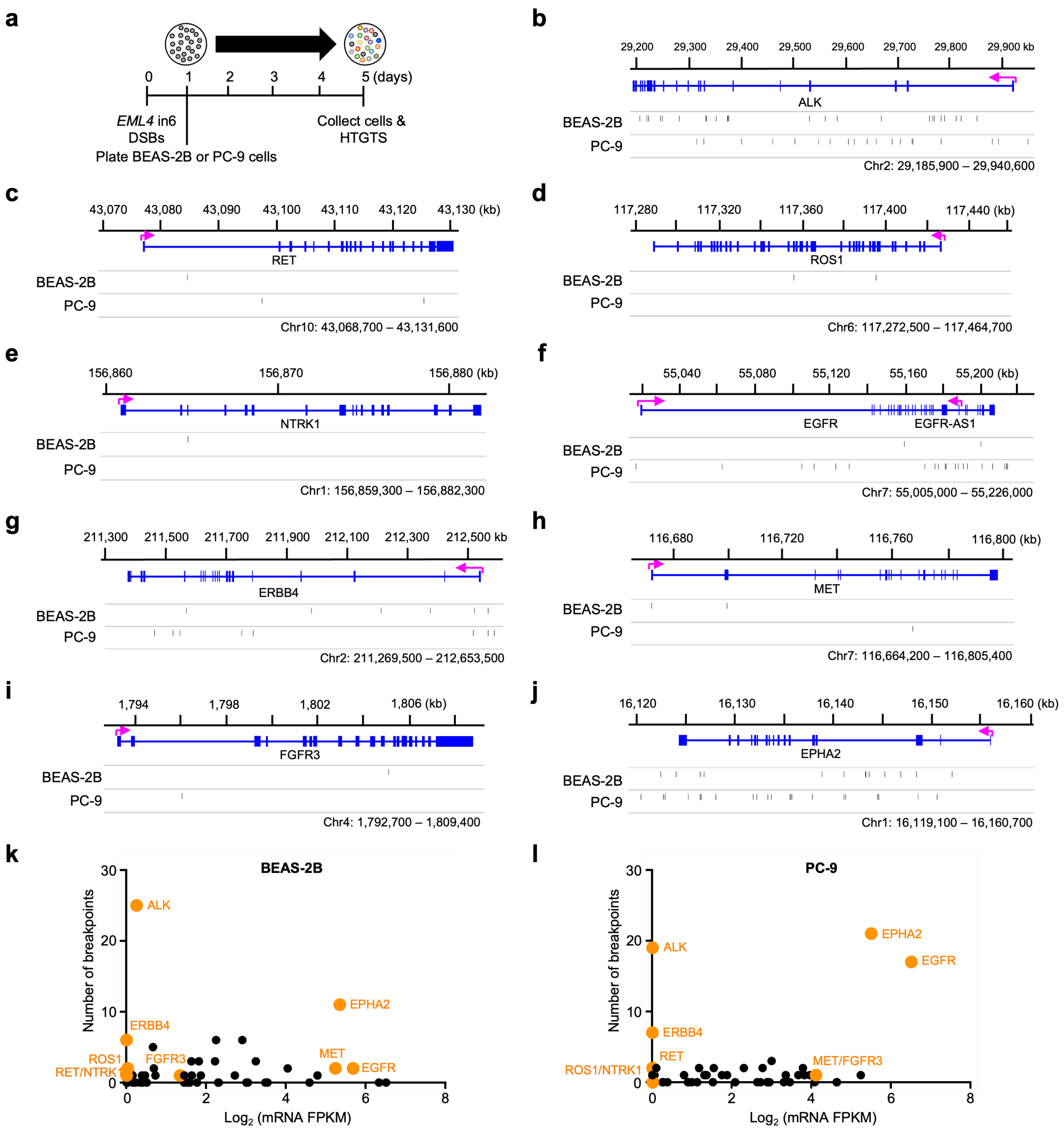

Supplementary Fig. 7. Characterization of translocation breakpoints in TK genes by HTGTS. a, Experimental timeline for HTGTS analysis introduced DSBs in *EML4*. **b-j**, Distribution of DNA breakpoints in *ALK* (**b**), *RET* (**c**), *ROS1* (**d**), *NTRK1* (**e**), EGFR (**f**), *ERBB4* (**g**), *MET* (**h**), *FGFR3* (**i**), *EPHA2* (**j**) TK genes in BEAS-2B and PC-9 cells introduced DSBs in intron 6 of *EML4*. **k and l**, Correlation between the number of DNA breakpoints and mRNA expression of TK genes in BEAS-2B (**k**) and PC-9 (**l**) cells. The TK genes showed in this figure are highlighted in cantaloupe. Source data are provided as a Source Data file.

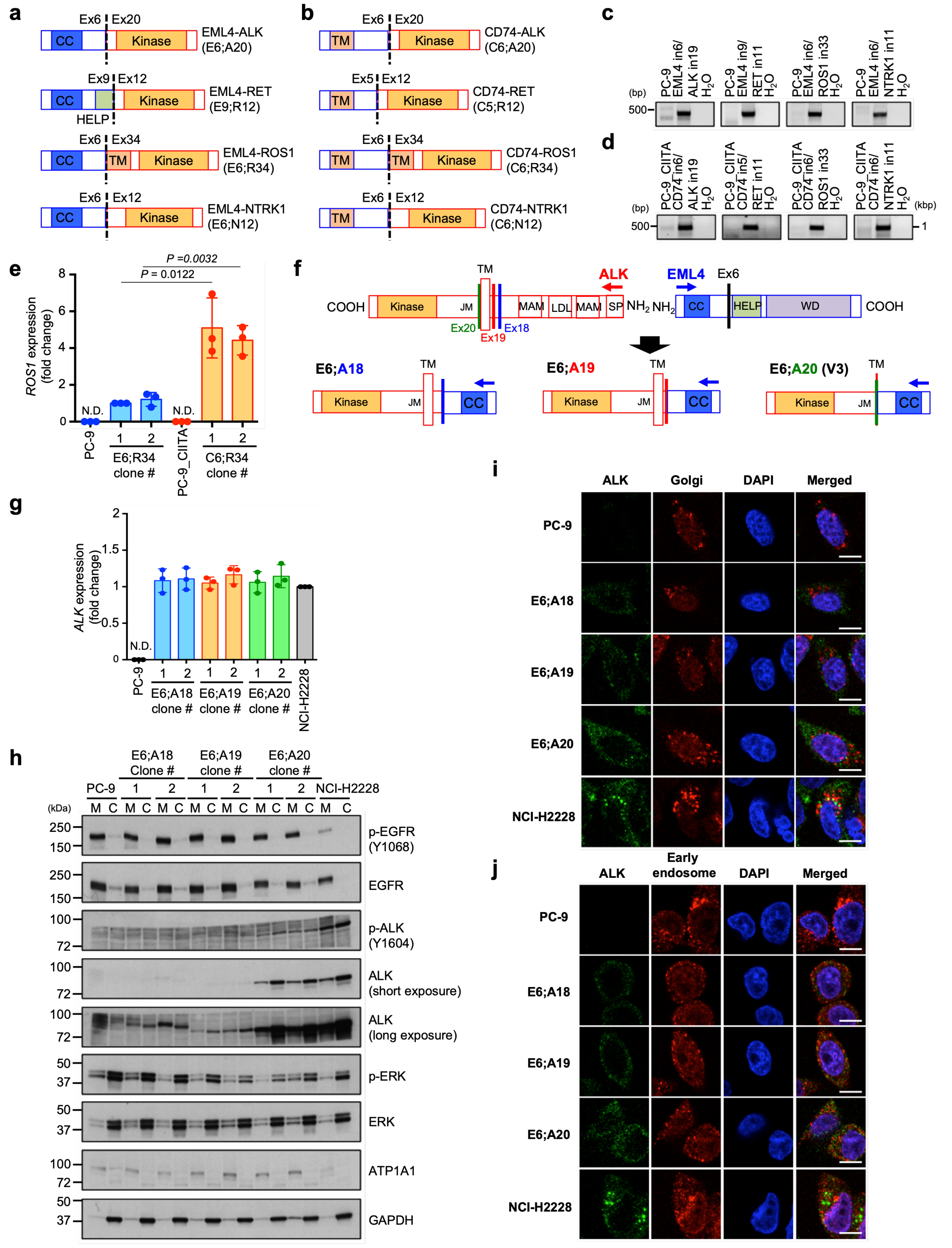

Supplementary Fig. 8. Characterization of EML4-TK, CD74-TK, and EML4-ALK fusion variants. a, Schematic structural composition of EML4-TK fusions. Protein domains are indicated by color and include: CC, coiled-coil domain; Kinase, tyrosine

kinase domain. **b**, Schematic structural composition of CD74-TK fusions. Protein domains are indicated by color and include: TM, transmembrane domain; Kinase, tyrosine kinase domain. **c and d**, Detection of EML4-TK (**c**) and CD74-TK (**d**) fusions by genomic DNA PCR. **e**, Messenger RNA expression in the cells expressing EML4-ROS1 or CD74-ROS1 fusion. Data show means of n=3 biological replicates, with error bars representing ±s.e.m; significance was determined by an unpaired, two-tailed Student's *t*-test. **f**, Schematic representation of EML4-ALK fusion variants. CC, coiled-coil domain; kinase, tyrosine kinase domain; TM, transmembrane domain. **g**, Expression of EML4-ALK transcripts by qRT-PCR. Data show means of n=3 biological replicates, with error bars representing ±s.e.m; significance was determined by an unpaired, two-tailed Student's *t*-test. **h**, Representative western blots of the signaling changes in membrane and cytosolic fractions in different EML4-ALK variants. Similar results were obtained from n=2 independent experiments. **i and j**, Subcellular localization of EML4-ALK variants in the Golgi (**i**) and the Early endosome (**j**). Scale bar = 10 μm. Similar results were obtained from n=3 independent experiments. Source data are provided as a Source Data file.

a

	Typical (n=97)	Atypical (n=11)
ALK breakpoint in	n (%)	n (%)
Intron 16		1 (9.1)
Intron 17		1 (9.1)
Intron 18		3 (27.3)
Exon 19		1 (9.1)
Intron 19	97 (100)	
Exon 20		3 (27.3)
Intron 20		2 (18.2)

b

Characteristic	Typical (n=97)	Atypical (n=11)	*p*-value
**Age**			
Mean (SD)	56.9 (11.5)	60.0 (14.9)	0.455
Median [Min, Max]	58.0 [23, 88]	60.0 [32, 83]	
**Sex**, n (%)			
Female	61 (62.9)	6 (54.5)	0.832
Male	36 (37.1)	5 (45.5)	
**Smoking history**, n (%)			
Ever	2 (2.1)	1 (9.1)	0.434
Former	19 (19.6)	3 (27.3)	
Never	71 (73.2)	7 (63.6)	
Current	5 (5.2)	0 (0)	
**Pack-years**			
Mean (SD)	2.67 (8.15)	8.91 (15.9)	0.162
Median [Min, Max]	0 [0, 50]	0 [0, 50]	
Missing	5 (5.2)	0 (0)	
**ECOG**, n (%)			
≥2	9 (11.5)	1 (9.1)	1
0-1	69 (88.5)	10 (90.9)	
Missing	19 (19.6)	0 (0)	
**Line of therapy**, n (%)			
First	77 (79.4)	9 (81.8)	1
Second or subsequent	20 (20.6)	2 (18.2)	
**ALK inhibitor**, n (%)			
Alectinib	66 (68.0)	6 (54.5)	0.507
Crizotininb	28 (28.9)	4 (36.4)	
Lorlatinib	2 (2.1)	1 (9.1)	
Ceritinib	1 (1.0)	0 (0)	

c

	Typical (n=97)	Atypical (n=11)
Partner gene	n (%)	n (%)
ARHGAP24	1 (1.0)	
CLTC		2 (18.2)
COX7A2L		1 (9.1)
DCTN1	1 (1.0)	
DNAH6	1 (1.0)	
EML4	86 (88.7)	6 (54.5)
ERC1		1 (9.1)
HIP1	1 (1.0)	
HIP1 and AUTS2	1 (1.0)	
KCNQ3	1 (1.0)	
PPP1R21		1 (9.1)
RP11-73M18.2	1 (1.0)	
Intergenic region	2 (2.1)	
Not mapped	2 (2.1)	

d

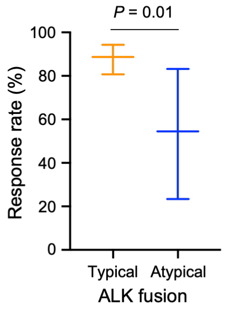

e

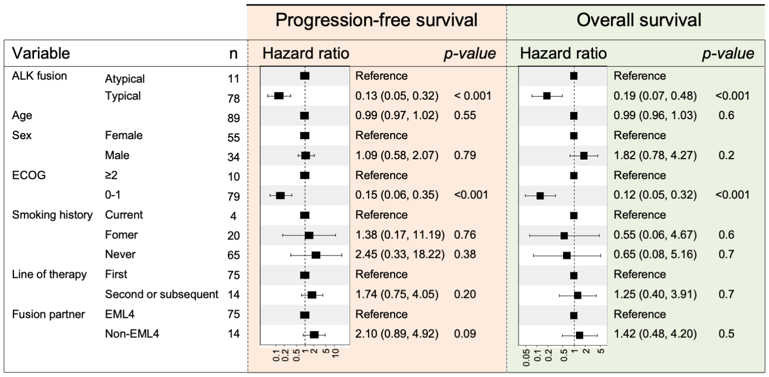

f

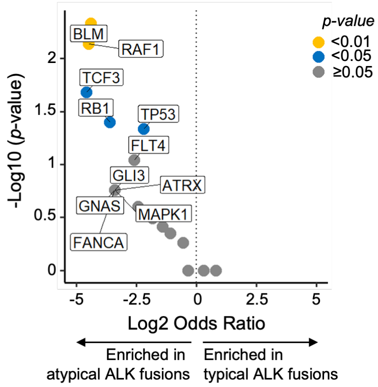

Supplementary Fig. 9. Detailed patient sample information for typical and atypical ALK fusions. a, Distribution of typical and atypical *ALK* fusions. **b**, Detailed characteristics of typical and atypical *ALK* fusion groups. **c**, Distribution of *ALK* fusion partner genes. **d**, Objective response rate to ALK TKI in typical and atypical groups. **e**, Multivariable Cox regression for progression-free survival (**middle**) and overall survival (**right**) to ALK TKI. **f**, Volcano plot showing gene mutations enriched in NSCLC with typical versus atypical ALK fusions. Source data are provided as a Source Data file.

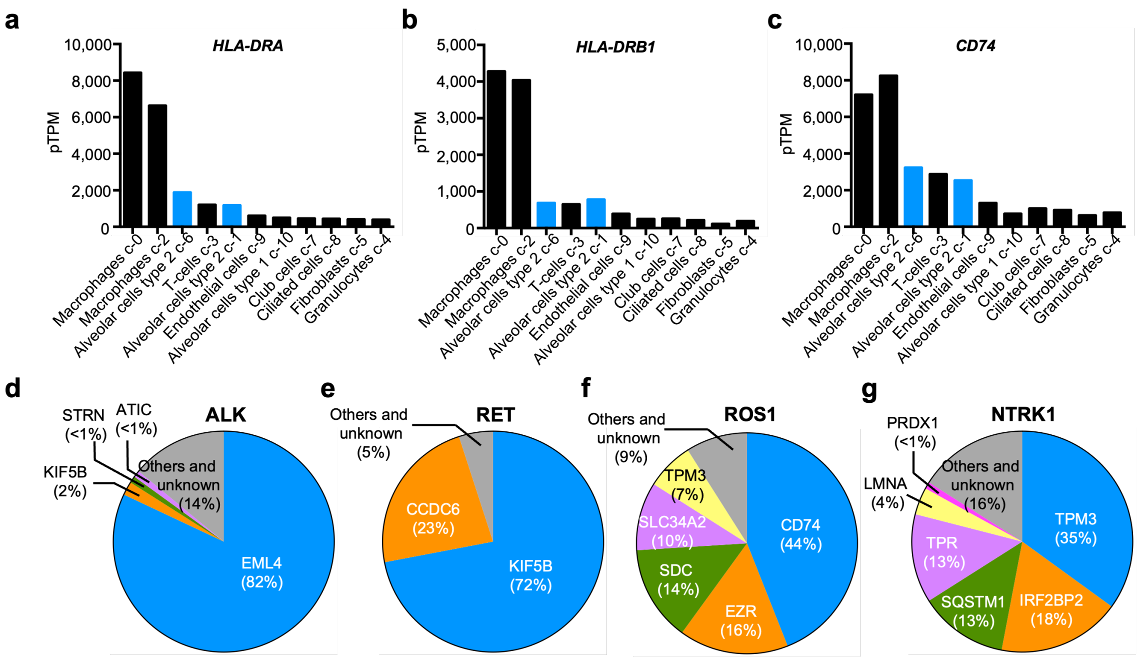

Supplementary Fig. 10. Frequencies of TK fusion partners in non-small cell lung cancers. a-c, Expression of *HLA-DRA* (**a**), *HLA-DRB1* (**b**), and *CD74* (**c**) from single-cell RNA-seq in the human lung. Data are adapted from the human protein atlas (http://www.proteinatlas.org). **d-g**, Frequencies of *ALK* (**d**), *RET* (**e**), *ROS1* (**f**), and *NTRK1* (**g**) fusion partners found in NSCLC patients. Data are modified from references[1-4].

Supplementary References

1. Noh, K.W., *et al.* Molecular breakdown: a comprehensive view of anaplastic lymphoma kinase (ALK)-rearranged non-small cell lung cancer. *J Pathol* **243**, 307-319 (2017).
2. Farago, A.F. & Azzoli, C.G. Beyond ALK and ROS1: RET, NTRK, EGFR and BRAF gene rearrangements in non-small cell lung cancer. *Transl Lung Cancer Res* **6**, 550-559 (2017).
3. Gainor, J.F. & Shaw, A.T. Novel targets in non-small cell lung cancer: ROS1 and RET fusions. *The oncologist* **18**, 865-875 (2013).
4. Xia, H., *et al.* Evidence of NTRK1 Fusion as Resistance Mechanism to EGFR TKI in EGFR+ NSCLC: Results From a Large-Scale Survey of NTRK1 Fusions in Chinese Patients With Lung Cancer. *Clinical lung cancer* **21**, 247-254 (2020).
