# Supplementary material for: Mechanistic patterns and clinical implications of oncogenic tyrosine kinase fusions in human cancers
Source: Nat Commun. 2024 Jun 14;15:5110. doi: 10.1038/s41467-024-49499-0 (PMC11178778; doi:10.1038/s41467-024-49499-0)
Supplement: Supplementary file 3 — Reporting Summary [file 41467_2024_49499_MOESM3_ESM.pdf]

nature portfolio

Corresponding author(s): Taek-Chin Cheong
Roberto Chiarle

Last updated by author(s): May 6, 2024

Reporting Summary

Nature Portfolio wishes to improve the reproducibility of the work that we publish. This form provides structure for consistency and transparency in reporting. For further information on Nature Portfolio policies, see our Editorial Policies and the Editorial Policy Checklist.

Statistics

For all statistical analyses, confirm that the following items are present in the figure legend, table legend, main text, or Methods section.

n/a	Confirmed	
☐	☒	The exact sample size (n) for each experimental group/condition, given as a discrete number and unit of measurement
☐	☒	A statement on whether measurements were taken from distinct samples or whether the same sample was measured repeatedly
☐	☒	The statistical test(s) used AND whether they are one- or two-sided<br>*Only common tests should be described solely by name; describe more complex techniques in the Methods section.*
☒	☐	A description of all covariates tested
☒	☐	A description of any assumptions or corrections, such as tests of normality and adjustment for multiple comparisons
☐	☒	A full description of the statistical parameters including central tendency (e.g. means) or other basic estimates (e.g. regression coefficient) AND variation (e.g. standard deviation) or associated estimates of uncertainty (e.g. confidence intervals)
☐	☒	For null hypothesis testing, the test statistic (e.g. F, t, r) with confidence intervals, effect sizes, degrees of freedom and P value noted<br>*Give P values as exact values whenever suitable.*
☒	☐	For Bayesian analysis, information on the choice of priors and Markov chain Monte Carlo settings
☒	☐	For hierarchical and complex designs, identification of the appropriate level for tests and full reporting of outcomes
☒	☐	Estimates of effect sizes (e.g. Cohen's d, Pearson's r), indicating how they were calculated

Our web collection on statistics for biologists contains articles on many of the points above.

Software and code

Policy information about availability of computer code

Data collection	HTGTS data were processed as previously described (Compagno M. et al, 2017). Gene fusions were obtained from Catalogue Of Somatic Mutations In Cancer (COSMIC, v98). ChIP-seq and RNA expression data were collected from public database: GEO, ENCODE and CGP.<br>For FACS: BD FACSCelesta using FACSDiva v8.0.1 software<br>For 3' end directed fusion assay: Archer Analysis v5.0.6 software<br>For sanger sequencing: MacVector v12.7.3 software
Data analysis	HTGTS data were analyzed as previously described (Compagno M. et al, 2017). Gene fusions were analyzed manually. ChIP-seq data were analyzed by BWA and MACS2, RNA-seq data were aligned by STAR v.2.6. Developed source codes are available at GitHub. Described more details in the Methods.<br>GraphPad Prism v7.03 were used for statistical analysis and graph visualization.<br>Flowjo v10.0.7 was used for analyzing the FACS data.<br>Archer analysis v5.0.6 software was used for 3' end directed fusion assay.<br>Integrative Genomics Viewer (IGV) v2.4.10 was used for all sequencing data analysis.<br>For genomic profiling analysis, Picard v1.90, MuTect v1.1.4, and gnomAD v.2.1.1 were used.<br><br>Source code for genomic event analysis tool (GEAT) developed in our laboratory to perform the analysis is available at http://github.com/geatools/geat.

For manuscripts utilizing custom algorithms or software that are central to the research but not yet described in published literature, software must be made available to editors and reviewers. We strongly encourage code deposition in a community repository (e.g. GitHub). See the Nature Portfolio guidelines for submitting code & software for further information.

Data

Policy information about availability of data

All manuscripts must include a data availability statement. This statement should provide the following information, where applicable:

- Accession codes, unique identifiers, or web links for publicly available datasets
- A description of any restrictions on data availability
- For clinical datasets or third party data, please ensure that the statement adheres to our policy

All sequencing data generated in this study have been deposited in the Gene Expression Omnibus (GEO) database under accession number GSE167155 [https://www.ncbi.nlm.nih.gov/geo/query/acc.cgi?acc=GSE167155]. RNA-seq data used in this study are available in the GEO database under accession number GSM4635290 [https://www.ncbi.nlm.nih.gov/geo/query/acc.cgi?acc=GSE153183]. ATAC-seq data used in this study are available in the GEO database under accession number GSM1904729 [https://www.ncbi.nlm.nih.gov/geo/query/acc.cgi?acc=GSM1904729]. H3K9ac ChIP-seq data used in this study are available in the GEO database under accession number GSM2534660 [https://www.ncbi.nlm.nih.gov/geo/query/acc.cgi?acc=GSM2534660]. H3K9me3 ChIP-seq data used in this study are available in the GEO database under accession number GSM1912806 [https://www.ncbi.nlm.nih.gov/geo/query/acc.cgi?acc=GSM1912806]. H3K4me3 ChIP-seq data used in this study are available in the Encyclopedia of DNA Elements (ENCODE) database under accession number ENCSR441JWF [https://www.encodeproject.org/experiments/ENCSR441JWF/]. H3K27ac ChIP-seq data used in this study are available in the ENCODE database under accession number ENCSR769FOC [https://www.encodeproject.org/experiments/ENCSR769FOC/]. Source data are provided with this paper.

Research involving human participants, their data, or biological material

Policy information about studies with human participants or human data. See also policy information about sex, gender (identity/presentation), and sexual orientation and race, ethnicity and racism.

Reporting on sex and gender	There is no individual level patient data presented.<br>Sex (biological attribute) was taken directly from the clinical chart review.<br>All data was de-identified.<br>Sex was used as a covariate in analysis of clinical data.
Reporting on race, ethnicity, or other socially relevant groupings	There is no race, ethnicity, or other socially relevant groupings.
Population characteristics	Clinical, pathologic, and outcomes data were abstracted via chart review. These included age, sex, smoking history, ECOG PS, histology. Genomic data were abstracted from tumor DNA sequencing data performed for clinical care.
Recruitment	Consecutive patients with advanced/metastatic ALK positive non-small lung cancer were included. Patients were treated as per standard of care. Patients were not recruited for participation.
Ethics oversight	Patients were included if they had consented to each institution's institutional review board-approved medical review protocols. The patient studies were conducted according to the ethical guidelines of the Declaration of Helsinki. De-identified patient data were used from patients who consented to IRB approved protocols Dana-Farber/Harvard Cancer Center 02-180, 11-104,13-364, and/or 17-000

Note that full information on the approval of the study protocol must also be provided in the manuscript.

Field-specific reporting

Please select the one below that is the best fit for your research. If you are not sure, read the appropriate sections before making your selection.

[x] Life sciences   [ ] Behavioural & social sciences   [ ] Ecological, evolutionary & environmental sciences

For a reference copy of the document with all sections, see nature.com/documents/nr-reporting-summary-flat.pdf

Life sciences study design

All studies must disclose on these points even when the disclosure is negative.

Sample size	No statistical methods were used to predetermine sample size for all experiments. Sample sizes were chosen based on previous studies in this field (Compagno M. et al, 2017) that used similar sample sizes to generate reproducible results.
Data exclusions	No data were excluded for statistical analysis.
Replication	All samples were analyzed by both biological and experimental repeats as detailed in the text, legends, and tables. All samples were analyzed with biological repeats. All data are reliably reproduced.
Randomization	Each experiment was performed with identified control. Randomization was not relevant the study as the study does not involve participant groups.
Blinding	No blinded group allocation was used during the experiment procedures. Blinding was not relevant to this study as each experiment was

associated with proper controls.

Reporting for specific materials, systems and methods

We require information from authors about some types of materials, experimental systems and methods used in many studies. Here, indicate whether each material, system or method listed is relevant to your study. If you are not sure if a list item applies to your research, read the appropriate section before selecting a response.

Materials & experimental systems

n/a	Involved in the study
☐	☒ Antibodies
☐	☒ Eukaryotic cell lines
☒	☐ Palaeontology and archaeology
☐	☒ Animals and other organisms
☐	☒ Clinical data
☒	☐ Dual use research of concern
☒	☐ Plants

Methods

n/a	Involved in the study
☒	☐ ChIP-seq
☐	☒ Flow cytometry
☒	☐ MRI-based neuroimaging

Antibodies

Antibodies used

ALK antibody (clone: D5F3, catalog number: 3633S, Cell Signaling Technology) was used for western blotting (1:2,000) and immonohistochemistry (1:100).
Phospho-ALK antibody (Tyr1604, catalog number: 3341S, Cell Signaling Technology) was used for western blotting (1:1,000).
EGF receptor antibody (catalog number: 2232, Cell signaling Technology) was used for western blotting (1:1,000).
Phospho-EGFR receptor antibody (Tyr1068, clone: D7A5, catalog number: 3777S, Cell Signaling Technology) was used for western blotting (1:1,000).
p44/42 MAPK (Erk1/2) antibody (clone: 137F5, catalog number: 4695S, Cell Signaling Technology) was used for western blotting (1:1,000).
Phospho-p44/42 MAPK (Erk1/2) antibody (Thr202/Tyr204, clone: D13.14.4E, catalog number: 4370, Cell Signaling Technology) was used for western blotting (1:1,000).
β-actin antibody (clone: 13E5, catalog number: 5125S, Cell Signaling Technology) was used for western blotting (1:2,000).
HLA-DR antibody (APC-conjugated, clone: C243, catalog number 340549, BD Biosciences) was used for flow cytometry (1:100).
CD74 antibody (PE-conjugated, clone: LN2, catalog number: 326808, BioLegend) was used for flow cytomerty (1:100).
CD74 antibody (PE-conjugated, clone: Pin.1, catalog number: 357604, BioLegend) was used for flow cytometry (1:100).
ROS1 antibody (clone: D4D6, catalog number: 63452S, Cell Signaling Technology) was used for western blotting (1:2,000).
Phospho-ROS1 antibody (Tyr2274, catalog number: 3078S, Cell Signaling Technology) was used for western blotting (1:1,000)
ATP1A1 antibody (clone: 464.6, catalog number: MA1-16731, Invitrogen) was used for western blotting. (1: 1,000)
ALK antibody (clone: 4C5B8, catalog number: 35-4300, Invitrogen) was used for immunofluorescence (1:200).
Golgin-97 antibody (clone: D8P2K, catalog number: 13192S, Cell Signaling Technology) was used for mmunofluorescence (1:200).
EEA1 antibody (clone: C45B10, catalog number: 3288, Cell Signaling Technology) was used for immunofluorescence (1:200).

Validation

ALK antibody (clone: D5F3, catalog number: 3633S, Cell Signaling Technology) was confirmed by western blotting by published paper including (except this study); Menotti M. et al., Wiskott-Aldrich syndrome protein (WASP) is a tumor suppressor in T cell lymphoma. Nature Medicine, 25(1):130-140. doi: 10.1038/s41591-081-0262-9 (2019).

Phospho-ALK antibody (Tyr1604, catalog number: 3341S, Cell Signaling Technology) was confirmed by western blotting by published paper including (except this study); Menotti M. et al., Wiskott-Aldrich syndrome protein (WASP) is a tumor suppressor in T cell lymphoma. Nature Medicine, 25(1):130-140. doi: 10.1038/s41591-081-0262-9 (2019).

EGF receptor antibody (catalog number: 2232, Cell signaling Technology) was confirmed by western blotting by published paper including (except this study); Tricker EM. et al., Combined EGFR/MEK inhibition prevents the emergence of resistance in EGFR-mutant lung cancer. Cancer Discovery, 5(9):960-971. doi: 10.1158/2159-8290.CD-15-0063 (2015).

Phospho-EGFR receptor antibody (Tyr1068, clone: D7A5, catalog number: 3777S, Cell Signaling Technology) was confirmed by western blotting by published paper including (except this study); Tricker EM. et al., Combined EGFR/MEK inhibition prevents the emergence of resistance in EGFR-mutant lung cancer. Cancer Discovery, 5(9):960-971. doi: 10.1158/2159-8290.CD-15-0063 (2015).

p44/42 MAPK (Erk1/2) antibody (clone: 137F5, catalog number: 4695S, Cell Signaling Technology) was confirmed by western blotting by published paper including (except this study); Menotti M. et al., Wiskott-Aldrich syndrome protein (WASP) is a tumor suppressor in T cell lymphoma. Nature Medicine, 25(1):130-140. doi: 10.1038/s41591-081-0262-9 (2019).

Phospho-p44/42 MAPK (Erk1/2) antibody (Thr202/Tyr204, clone: D13.14.4E, catalog number: 4370, Cell Signaling Technology) was confirmed by western blotting by published paper including (except this study); Menotti M. et al., Wiskott-Aldrich syndrome protein (WASP) is a tumor suppressor in T cell lymphoma. Nature Medicine, 25(1):130-140. doi: 10.1038/s41591-081-0262-9 (2019).

β-actin antibody (clone: 13E5, catalog number: 5125S, Cell Signaling Technology) is validated by he manufacture. The manufacture's

website is following; https://www.cellsignal.com/products/antibody-conjugates/b-actin-13e5-rabbit-mab-hrp-conjugate/5125.

The flow cytometry antibodies were validated by using corresponding negative cells for each stains.
HLA-DR antibody (APC-conjugated, clone: C243, catalog number 340549, BD Biosciences), CD74 antibody (PE-conjugated, clone: LN2, catalog number: 326808, BioLegend), CD74 antibody (PE-conjugated, clone: Pin.1, catalog number: 357604, BioLegend) were validated in our lab.

ROS1 antibody (clone: D4D6, catalog number: 63452S, Cell Signaling Technology) was confirmed by western blotting by published paper including; Rimkunas VM. et al., Analysis of receptor tyrosine kinase ROS1-positive tumors in non-small cell lung cancer: identification of a FIG-ROS fusion. Clinical Cancer Research, 18(16):4449-57. doi: 10.1158/1078-0432.CCR-11-3351. (2012).

Phospho-ROS1 antibody (Tyr2274, catalog number: 3078S, Cell Signaling Technology) was confirmed by western blotting by published paper including; Liu Z. et al., ROS1-fusion protein induces PD-L1 expression via MEK-ERK activation in non-small cell lung cancer. Oncoimmunology, 9(1):1758003. doi: 10.1080/2162402X.2020.1758003. (2020).

ATP1A1 antibody (clone: 464.6, catalog number: MA1-16731, Invitrogen)was confirmed by western blotting by published paper including; Smith RS. et al., Sodium channel SCN3A (Nav1.3) regulation of human cerebral cortical folding and oral motor development. Neuron, 99(5):905-913.e7. doi: 10.1016/j.neuron.2018.07.052. (2018).

ALK antibody (clone: 4C5B8, catalog number: 35-4300, Invitrogen)was confirmed by western blotting by published paper including; Ceccon M. et al., Excess of NPM-ALK oncogenic signaling promotes cellular apoptosis and drug dependency. Oncogene, 35 (29):3854-3865. doi: 10.1038/onc.2015.456. (2016)

Golgin-97 antibody (clone: D8P2K, catalog number: 13192S, Cell Signaling Technology) was confirmed by western blotting by published paper including; Bannoud N et al., Cation-dependent mannose-6-phosphate receptor expression and distritubion are influenced by estradiol in MCF-7 breast cancer cells. PLoS One, 13(8):e0201844. doi: 10.1371/journal.pone0201844. (2018)

EEA1 antibody (clone: C45B10, catalog number: 3288, Cell Signaling Technology) was confirmed by western blotting by published paper including; Tulpule A. et al., Kinase-mediated RAS signaling via membraneless cytoplasmic protein granules. Cell, 184 (10):2649-2664.e18. doi: 10.1016/j.cell.2021.03.031. (2021)

Eukaryotic cell lines

Policy information about cell lines and Sex and Gender in Research

Cell line source(s)	PC-9 cells were a kind gift from Dr. Pasi A. Jänne (Dana-Farber Cancer Institute, Boston, MA; Millipore Sigma, catalog number 90071810).<br>BEAS-2B cells were obtained from the ATCC (catalog number: CRL-3588).<br>293FT cells were obtained from ThermoFisher Scientific (catalog number: R70007).<br>NCI-H2228 (CRL-5935) was obtained from ATCC.<br>NCI-H3122 (300484) was obtained from Cytion.
Authentication	All the cell lines were obtained from the ATCC and ThermoFisher Scientific, no additional authentication performed. Cells were aliquoted in frozen vials and were used within two months after thawing.<br>Only NCI-H2228 and NCI-H3122 cell lines were further authenticated by western blotting.
Mycoplasma contamination	All cell lines were tested negative for mycoplasma contamination.
Commonly misidentified lines<br>(See ICLAC register)	No commonly misidentified cell lines were used in the study.

Animals and other research organisms

Policy information about studies involving animals; ARRIVE guidelines recommended for reporting animal research, and Sex and Gender in Research

Laboratory animals	We used immunodeficient NOD SCID gamma (NSG) mice (NOD.Cg-Prkdcscid Il2rgtm1Wjl/SzJ, Stock number: 005557; The Jackson Laboratory) for xenograft experiments. We used both male and female mice at the ages of 6-12 weeks.
Wild animals	This study did not involve wild animals.
Reporting on sex	We used 48 NSG mice in total and involved both male and female in this study. Sex-based analyses were not performed.
Field-collected samples	This study did not employed field-collected samples.
Ethics oversight	All mouse works were performed in compliance with all the relevant ethical regulations established by the Institutional Animal Care and Use Committee (IACUC) of Boston Children' Hospital and under the mouse protocol approved by the IACUC of Boston Children's Hospital protocol #00001530). The maximal tumor size permitted was 1.5 cm in maximum diameter for tumors. The maximal tumor size was never exceeded.

Note that full information on the approval of the study protocol must also be provided in the manuscript.

Clinical data

Policy information about clinical studies

All manuscripts should comply with the ICMJEguidelines for publication of clinical research and a completedCONSORT checklist must be included with all submissions.

Clinical trial registration	N/A. Patients were treated with standard of care therapies.
Study protocol	Institutional review board (IRB) at Dana-Farber Cancer Institute (DFCI) approved protocols Dana-Farber/Harvard Cancer Center (DF/HCC) 02-180, 11-104,13-364, and/or 17-000.
Data collection	Clinical chart review was used for data collection. Consecutive patients from 2009 to 2023 were included.
Outcomes	Endpoint point included objective repose rate, progression-free survival and overall survival to ALK TKIs. Thus were assessed by physician review and clinical chart review. A description of the covariate relevant study population and tumor characteristics including age, line of treatment, and other clinical characteristics can be found in Supplementary Data 8.

Plants

Seed stocks	*Report on the source of all seed stocks or other plant material used. If applicable, state the seed stock centre and catalogue number. If plant specimens were collected from the field, describe the collection location, date and sampling procedures.*
Novel plant genotypes	*Describe the methods by which all novel plant genotypes were produced. This includes those generated by transgenic approaches, gene editing, chemical/radiation-based mutagenesis and hybridization. For transgenic lines, describe the transformation method, the number of independent lines analyzed and the generation upon which experiments were performed. For gene-edited lines, describe the editor used, the endogenous sequence targeted for editing, the targeting guide RNA sequence (if applicable) and how the editor was applied.*
Authentication	*Describe any authentication procedures for each seed stock used or novel genotype generated. Describe any experiments used to assess the effect of a mutation and, where applicable, how potential secondary effects (e.g. second site T-DNA insertions, mosiacism, off-target gene editing) were examined.*

Flow Cytometry

Plots

Confirm that:

- [x] The axis labels state the marker and fluorochrome used (e.g. CD4-FITC).
- [x] The axis scales are clearly visible. Include numbers along axes only for bottom left plot of group (a 'group' is an analysis of identical markers).
- [x] All plots are contour plots with outliers or pseudocolor plots.
- [x] A numerical value for number of cells or percentage (with statistics) is provided.

Methodology

Sample preparation	PC-9 cells were transfected with pcDNA myc CIITA (P#808) plasmid (Addgene plasmid #14650), were selected, and sorted by staining with APC-conjugated HLA antibody (clone:3243, catalog number: 340549, BD Bioscience).
Instrument	Cells were analyzed on a BD FACSCelesta (BD Biosciences) and were sorted by a FACSAria II (BD Biosciences).
Software	All data were collected via FACSDiva v8.0.1. software (BD Biosciences) and were analyzed by FlowJo v10.0.7 (Tree Star).
Cell population abundance	Purity of cell populations of HLA-DR+ PC-9 cells was assessed by post-sort analysis on the FACSCelesta; purity of over 85% was achieved.
Gating strategy	Live cell population was gated by forward (FSC) and side (SSC) scatters out of the total cells. Single cells were gated from live cells by excluding doublet cells by using a forward scatter height (FSC-H) vs. forward scatter area (FSC-A) density plot. HLA-DR and CD74 expression levels were plotted from the single cells. Gating strategy for flow cytometry was provided in the Source Data file.

- [x] Tick this box to confirm that a figure exemplifying the gating strategy is provided in the Supplementary Information.
