# Supplementary material for: Mechanistic patterns and clinical implications of oncogenic tyrosine kinase fusions in human cancers
Source: Nat Commun. 2024 Jun 14;15:5110. doi: 10.1038/s41467-024-49499-0 (PMC11178778; doi:10.1038/s41467-024-49499-0)
Supplement: Supplementary file 4 — Description of Additional Supplementary Files [file 41467_2024_49499_MOESM4_ESM.pdf]

Description of Additional Supplementary Files

Supplementary Data 1. The number of kinase fusions in COSMIC v98

Supplementary Data 2. The distribution of kinase fusions across tissue types in COSMIC v98

Supplementary Data 3. The distribution of kinase fusions in COSMIC v98

Supplementary Data 4. Functional fusion partners of ALK, RET, ROS1, and NTRK1 identified in PC-9 cells by FACTS

Supplementary Data 5. Functional fusion partners of ROS1 identified in CIITA-expressing PC-9 cells by FACTS

Supplementary Data 6. Hotspot list for before and after osimertinib selection identified by HTGTS

Supplementary Data 7. RNA abundance and the number of DSBs in TK genes in BEAS-2B and PC-9 cells

Supplementary Data 8. Detailed information for patient samples

Supplementary Data 9. Oligonucleotides used in this study
